# Supplementary material for: Investigating the oxidative stress–vascular brain injury axis in mild cognitive impairment of the Alzheimer's type
Source: Alzheimers Dement. 2025 Sep 15;21(9):e70456. doi: 10.1002/alz.70456 (PMC12434610; doi:10.1002/alz.70456)
Supplement: Supplementary file 3 — Supporting Information [file ALZ-21-e70456-s001.docx]

Supplementary Table S2: Comparison of metabolite levels in individuals with low GSH (below the median) and high GSH (above or equal to the median), and comparison of metabolite ratios in individuals with with low GSH/tCr (below the median) and high GSH/tCr (above or equal to the median). *Abbreviations*: GSH, glutathione; tNAA total N-acetylaspartate; Glu, glutamate; Gln, glutamine; Glx, glutamate + glutamine; tCho, total choline-containing compounds; mI, myo-inositol; tCr, total creatine; PCC, posterior cingulate cortex; FWM, frontal white matter.

| **ROI** | **Group** | **GSH** | | **tNAA** | | **Glu** | | **Gln** | | **Glx** | | **tCho** | | **mI** | | **tCr** | |
| --- | --- | --- | --- | --- | --- | --- | --- | --- | --- | --- | --- | --- | --- | --- | --- | --- | --- |
|  |  | mean | *p* | mean | *p* | mean | *p* | mean | *p* | mean | *p* | mean | *p* | mean | *p* | mean | *p* |
| PCC | Low GSH | 1.47 | **< 0.001** | 14.27 | 0.19 | 10.06 | 0.54 | 2.19 | 0.15 | 12.25 | 0.30 | 1.92 | **= 0.05** | 9.26 | 0.08 | 10.25 | 0.25 |
|  | High GSH | 1.75 |  | 14.90 |  | 10.34 |  | 2.42 |  | 12.76 |  | 2.11 |  | 10.02 |  | 10.51 |  |
| FWM | Low GSH | 1.41 | **< 0.001** | 10.13 | 0.11 | 5.66 | 0.76 | 1.59 | 0.64 | 7.25 | 0.94 | 2.65 | 0.14 | 8.67 | 0.91 | 7.97 | 0.65 |
|  | High GSH | 1.77 |  | 10.66 |  | 5.73 |  | 1.49 |  | 7.22 |  | 2.89 |  | 8.61 |  | 8.05 |  |
| **ROI** | **Group** | **GSH/tCr** | | **tNAA/tCr** | | **Glu/tCr** | | **Gln/tCr** | | **Glx/tCr** | | **tCho/tCr** | | **mI/tCr** | | **tCr/tCr** | |
|  |  | mean | *p* | mean | *p* | mean | *p* | mean | *p* | mean | *p* | mean | *p* | mean | *p* | mean | *p* |
| PCC | Low GSH/tCr | 0.14 | **< 0.001** | 1.38 | **< 0.05** | 0.98 | 0.86 | 2.19 | 0.56 | 1.20 | 0.61 | 0.19 | 0.48 | 0.91 | 0.26 | — | |
|  | High GSH/tCr | 0.17 |  | 1.43 |  | 0.99 |  | 0.23 |  | 1.22 |  | 0.20 |  | 0.95 |  |  |  |
| FWM | Low GSH/tCr | 0.17 | **< 0.001** | 1.26 | **< 0.05** | 0.70 | 0.39 | 0.19 | 0.84 | 0.89 | 0.64 | 0.33 | 0.12 | 1.07 | 0.95 |  |  |
|  | High GSH/tCr | 0.22 |  | 1.34 |  | 0.73 |  | 0.19 |  | 0.91 |  | 0.36 |  | 1.07 |  |  |  |
